# Host-specific thermal profiles affect fitness of a widespread pathogen

**DOI:** 10.1002/ece3.1271

**Published:** 2014-10-03

**Authors:** Lisa A Stevenson, Elizabeth A Roznik, Ross A Alford, David A Pike

**Affiliations:** School of Marine and Tropical Biology, James Cook UniversityTownsville, Queensland, Australia

**Keywords:** Amphibian chytrid fungus, amphibian decline, *Batrachochytrium dendrobatidis*, body temperature, *Litoria nannotis*, *Litoria rheocola*, *Litoria serrata*, thermal behavior

## Abstract

Host behavior can interact with environmental context to influence outcomes of pathogen exposure and the impact of disease on species and populations. Determining whether the thermal behaviors of individual species influence susceptibility to disease can help enhance our ability to explain and predict how and when disease outbreaks are likely to occur. The widespread disease chytridiomycosis (caused by the fungal pathogen *Batrachochytrium dendrobatidis*, *Bd*) often has species-specific impacts on amphibian communities; some host species are asymptomatic, whereas others experience mass mortalities and population extirpation. We determined whether the average natural thermal regimes experienced by sympatric frog species in nature, in and of themselves, can account for differences in vulnerability to disease. We did this by growing *Bd* under temperatures mimicking those experienced by frogs in the wild. At low and high elevations, the rainforest frogs *Litoria nannotis*, *L. rheocola,* and *L. serrata* maintained mean thermal regimes within the optimal range for pathogen growth (15–25°C). Thermal regimes for *L. serrata*, which has recovered from *Bd*-related declines, resulted in slower pathogen growth than the cooler and less variable thermal regimes for the other two species, which have experienced more long-lasting declines. For *L. rheocola* and *L. serrata*, pathogen growth was faster in thermal regimes corresponding to high elevations than in those corresponding to low elevations, where temperatures were warmer. For *L. nannotis*, which prefers moist and thermally stable microenvironments, pathogen growth was fastest for low-elevation thermal regimes. All of the thermal regimes we tested resulted in pathogen growth rates equivalent to, or significantly faster than, rates expected from constant-temperature experiments. The effects of host body temperature on *Bd* can explain many of the broad ecological patterns of population declines in our focal species, via direct effects on pathogen fitness. Understanding the functional response of pathogens to conditions experienced by the host is important for determining the ecological drivers of disease outbreaks.

## Introduction

Epidemic disease can drive rapid population declines that can lead to extinction in some species, despite the persistence of other sympatric but also susceptible species. Disease emergence can be triggered by changes in the ecology of the host and/or pathogen through stressors such as climate change and habitat disturbance, and epidemics can then spread rapidly through populations (Harvell et al. 2002; Kiesecker 2011). The population-level processes that control the outcomes of epidemics can be summarized by epidemiological models that describe fractions of the population as follows: Susceptible to infection (S), Exposed to infection (E), Infective to other individuals (I), or Recovered (R, i.e., immune) (Hethcote 1976; Liu et al. 1987; deCastro and Bolker 2005). How individuals move among these states, and whether infection is fatal, can depend on host behavior (Rowley and Alford 2007a, 2013; Richards-Zawacki 2010; Daly and Johnson 2011) and on both innate and adaptive immune defenses (Rollins-Smith et al. 2002; Woodhams et al. 2007a; Melzer and Bishop 2010). Determining the relative influence of behavior on the functional response of pathogens will enhance our ability to predict how and when disease outbreaks are likely to occur.

Many contemporary amphibian declines have been caused by the emergence of the disease chytridiomycosis (Berger et al. 1998). Chytridiomycosis is caused by the fungal pathogen *Batrachochytrium dendrobatidis* (*Bd*), which is presently known to infect over 350 amphibian species (Fisher et al. 2009). Extinctions are usually not associated with disease because epidemics often fade out when host populations fall below the density required for transmission (deCastro and Bolker 2005). Chytridiomycosis is an exception to this rule because it can drive some host species to complete local extinction (Berger et al. 1998; Lips et al. 2006; Rachowicz et al. 2006), perhaps in part because less vulnerable species serve as reservoirs (Daszak et al. 2004; Vrendenburg et al. 2010; Reeder et al. 2012). For example, in Australia, the sharp-snouted day frog *Taudactylus acutirostris* (Schloegel et al. 2006) and northern gastric brooding frog *Rheobatrachus vitellinus* (Retallick et al. 2004) have gone extinct, probably due to chytridiomycosis, while other frogs within the same habitats have been less affected by the pathogen (Richards et al. 1993; McDonald and Alford 1999).

*Batrachochytrium dendrobatidis* is extremely sensitive to temperature and requires relatively cool (≤26–28°C, depending on the isolate), moist conditions to survive and reproduce (Piotrowski et al. 2004; Stevenson et al. 2013). Frog species that are more restricted to moist, cool conditions, such as stream-associated species at high elevations, are thus more vulnerable to chytridiomycosis-related declines (Bielby et al. 2008; Bancroft et al. 2011). It is becoming apparent that understanding the behavior, ecology, and physiology of host species is crucial to understanding patterns of vulnerability to disease, because the outcome of *Bd* infection in the wild is influenced by species-specific host behavior (microhabitat use, thermal preferences, and social behaviors; Rowley and Alford 2007a; Richards-Zawacki 2010; Daskin et al. 2011; Rowley and Alford 2013; Roznik 2013), by skin peptides (Woodhams et al. 2007b) and by skin microbes (Rollins-Smith et al. 2002; Harris et al. 2006, 2009; Melzer and Bishop 2010). These factors often interact, with the effects of one factor depending at least in part on those of another (Woodhams et al. 2007b; Rowley and Alford 2010). For example, because many aspects of species defenses covary with patterns of body temperature variation (Maniero and Carey 1997; Raffel et al. 2006; Ribas et al. 2009; Daskin et al. 2014), separating the effects of body temperature on the growth of *Bd* from other species-specific factors cannot be performed in living frogs. A more complete understanding of the role of temperature alone in influencing rates of pathogen development will help determine the extent to which environmental temperatures and species-specific body temperature patterns produced by behavioral differences influence pathogen life history.

We sought to clarify the extent to which host-specific body temperatures directly influence susceptibility to disease. We accomplished this by incubating *Bd* cultures in fluctuating thermal regimes that mimicked in detail the average thermal regimes experienced by frogs in nature. Previous studies that have incubated *Bd* under fluctuating thermal conditions have used arbitrary means and variances, rather than regimes based on data collected from populations of particular species in the field. We compared the growth and reproductive output of *Bd* incubated in vitro under conditions emulating the mean thermal regimes of three rainforest frog species (*Litoria nannotis*, *L. rheocola* and *L. serrata*) from high- and low-elevation populations. These species are stream-associated rainforest specialists with broadly overlapping distributions in the Wet Tropics region of northern Queensland, Australia (Williams 2006) that differ substantially in their ecology, behavior, and thermal relations (Rowley and Alford 2007a, 2013; Roznik 2013). The waterfall frog *L. nannotis* is truly stream dwelling; both males and females spend much of the day sheltering behind waterfalls or wedged between rocks in the stream, and do not often venture far from streams, even at night (Hodgkison and Hero 2001; Rowley and Alford 2007a; Puschendorf et al. 2012). By contrast, the green-eyed treefrog *L. serrata* reproduces in streams, but can often be found in or on surrounding vegetation (Richards and Alford 2005; Rowley and Alford 2007a). The common mistfrog *L. rheocola* also reproduces in streams, with males being more tightly associated with streams than male *L. serrata* (McDonald and Alford 1999; Roznik 2013); it is thus intermediate between *L. nannotis* and *L. serrata*. The histories of population declines differ among these species; *L. serrata* experienced short-term declines at high (>400 m) elevation sites when *Bd* first emerged but is now common at many sites, while *L. nannotis* and *L. rheocola* experienced extirpation at all known high-elevation rainforest sites (Richards et al. 1993; McDonald and Alford 1999).

We predicted that the in vitro growth patterns of *Bd* would differ among the thermal regimes of these species, with lower *Bd* growth in the thermal regimes of *L. serrata* (which underwent short-term declines) than in those of *L. nannotis* and *L. rheocola* (which experienced more severe declines and local extirpations). We also predicted that *Bd* would perform better in vitro in the high-elevation thermal regimes than in the low-elevation regimes and that growth patterns in fluctuating temperature regimes, would differ from growth patterns in constant-temperature regimes with the same mean (Georges 1989).

## Materials and Methods

### Natural thermal data

We obtained detailed data on the natural thermal regimes of three frog species endemic to the Wet Tropics region of Queensland, Australia: The waterfall frog *Litoria nannotis*, the common mistfrog *L. rheocola*, and the green-eyed treefrog *L. serrata*. Because infection by *Bd* may influence frog behavior (Richards-Zawacki 2010; Roznik 2013), we only used thermal regimes from male frogs that tested negative for *Bd* infection via diagnostic quantitative PCR (Boyle et al. 2004). This allowed us to assess how thermal regimes may contribute to the vulnerability of males of each species becoming infected, in isolation from other species-specific factors such as innate and adaptive immune responses, changes in behavior in response to infection, or skin microbiota. Thermal data were collected during winter (the cool/dry season) at one low- and one high-elevation site for each species (Appendix S1). The high-elevation study site was the same for all three species (Windin Creek, Wooroonooran National Park; 17.365°S, 145.717°E, 750 m). Study periods at Windin Creek were June 20–July 04, 2010 for *L. nannotis* (*N* = 7), August 18–September 09, 2009 for *L. rheocola* (*N* = 7), and August 26–September 08, 2011 for *L. serrata* (*N* = 11). The low-elevation sites differed among species; we studied *L. nannotis* (*N* = 10) at Kirrama Creek 8 in Girramay National Park (June 15–19, 2010; 18.196°S, 145.868°E, 170 m), *L. rheocola* (*N* = 19) at Frenchman Creek in Wooroonooran National Park (July 13–August 06, 2009; 17.307°S, 145.922°E, 40 m), and *L. serrata* (*N* = 14) at Stoney Creek in Djiru National Park (August 12–25, 2011; 17.920°S, 146.069°E, 20 m). These threatened species have all declined due to chytridiomycosis and thus are unavailable for use in manipulative laboratory experiments.

We used two methods to collect thermal data from frogs (Roznik 2013). For *L. serrata*, we attached temperature-sensitive radiotransmitters (Model A2414; Advanced Telemetry Systems, Isanti, MN) to each individual. Transmitters emitted signals in pulses at rates that depended on temperature. The pulse rate of each transmitter was recorded by an automated data logger receiver (Model SRX400A; Lotek Wireless, Newmarket, ON, Canada) every 15 min during the study period and was later converted to temperature using calibration curves provided for each transmitter by the manufacturer. We used this dataset to generate thermal regimes for this experiment by calculating the median temperature at each 15-min interval throughout the 24-h day for each individual frog, and then averaging these temperatures for all frogs at each 15-min interval.

**Figure 1:**
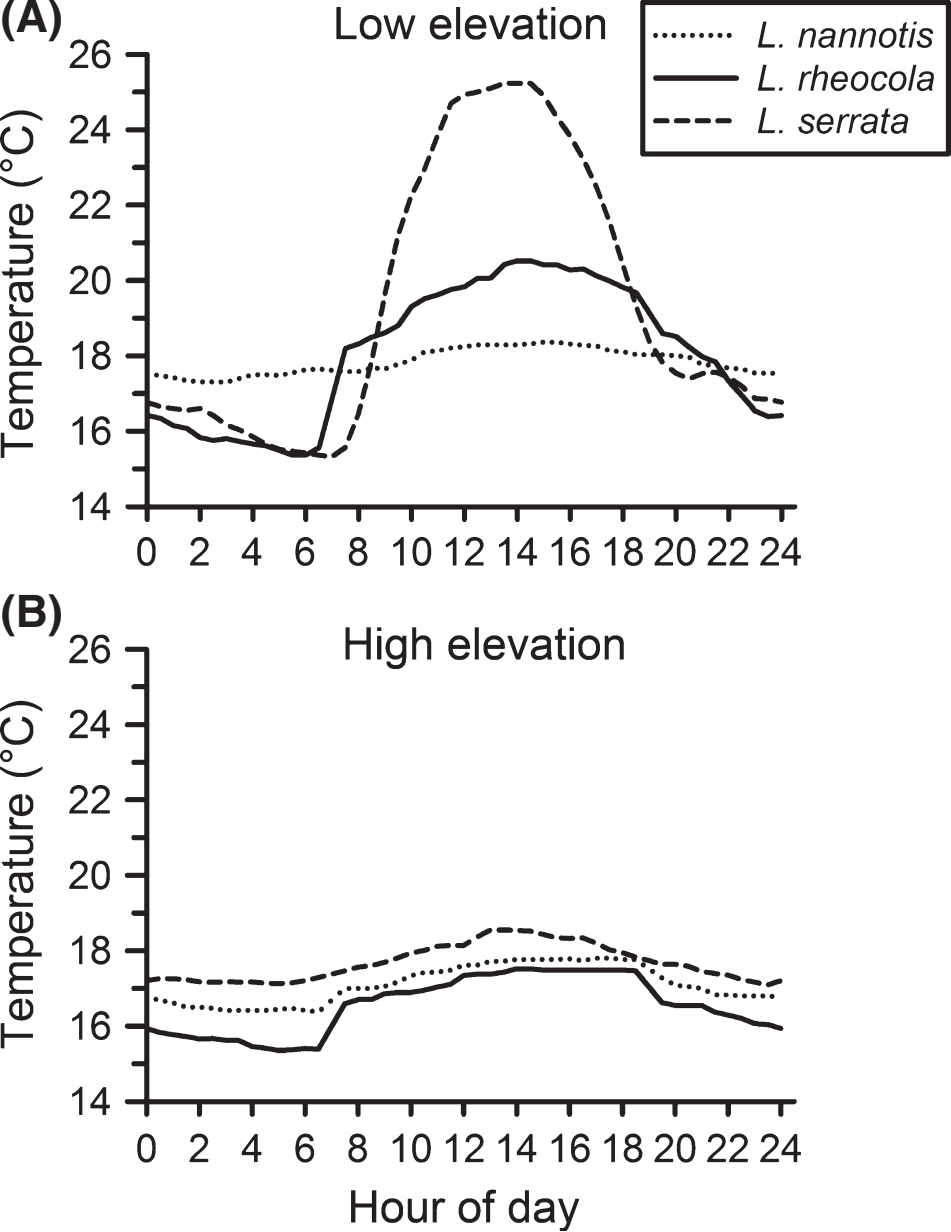
Natural thermal regimes from (A) low- and (B) high-elevation sites for the frogs *Litoria nannotis* (*N* = 10 and 7 frogs, respectively), *L. rheocola* (*N* = 19 and 7 frogs), and *L. serrata* (*N* = 14 and 11 frogs) during the cool/dry winter season. The daily mean temperatures of frogs at low and high elevations were 17.8 and 17.1°C for *L. nannotis*, 16.6 and 18.1°C for *L. rheocola*, and 19.4 and 17.7°C for *L. serrata*. Daily median temperatures were within ±0.3°C of these means, except for *L. serrata* at the low elevation site, which had a lower median of 17.6°C.

For *L. nannotis* and *L. rheocola*, we collected thermal data from physical models placed in locations used by frogs (Rowley and Alford 2010; Roznik and Alford 2014). To locate frogs, *L. nannotis* was tracked using radiotelemetry, and *L. rheocola* was tracked using harmonic direction-finding (Langkilde and Alford 2002; Rowley and Alford 2007b). Both species were tracked during the day (10:00–17:00 h) and again at night (20:00–03:00 h), and physical models were placed in each unique location used by each frog (Roznik 2013). Physical models consisted of paired frog models made of three percent agar, each embedded with a Thermochron iButton temperature logger (Maxim Integrated Products, CA; factory-calibrated and accurate to ±0.5°C) programmed to record temperatures at 30-min intervals (Rowley and Alford 2010). Model pairs comprised one model that was permeable to water loss, whereas the other was coated with plastic to prevent water loss. Together, these models can be used to define the upper and lower boundaries of possible amphibian body temperatures at the locations used by frogs, and the temperatures they measure are highly correlated with actual frog body temperatures collected using more direct methods (Rowley and Alford 2010; Roznik and Alford 2014). We placed models in daytime locations used by frogs to measure temperatures between 07:00 and 18:30 h, and placed models in night-time locations used by frogs to measure temperatures between 19:00 and 06:30 h. To generate thermal regimes for each species/elevation combination, we first calculated the median value at each 30-min interval for each model type (impermeable and permeable to water loss) for each individual frog. This provided the upper and lower boundaries of possible body temperatures, which were averaged for individual frogs at each 30-min interval. To obtain our final thermal regimes, we averaged these thermal regimes across all individual frogs at each 30-min interval for each elevation/species combination (Fig. 1).

### Recreating natural thermal regimes

Our objective was to determine how the natural thermal regimes of uninfected male frogs of three species would affect the growth rate of *Bd* in isolation from all other aspects of the host-pathogen system. To do this, we recreated the thermal regimes of each species at both low and high elevations in six temperature-controlled incubators (accuracy and precision to ±0.5°C). To program the incubators, we divided our final 24-h thermal cycles into 4-h intervals; we used the average maximum value from each interval to generate simplified thermal cycles that maintained daily thermal variation and closely mimicked the full thermal regimes (±0.5°C). For simplicity, we refer to each of the six thermal treatments using the elevation and species name (e.g., the *L. serrata* regime at high elevation as “High-Serrata”). During the experiments, we recorded temperatures within each incubator every 15 min using Thermochron iButton data loggers (two per incubator). Full replication of the thermal regimes (*N* = 6) was not possible due to the limited number of incubators available (*n* = 10). While a number of factors are known to influence *Bd* growth (i.e., temperature, desiccation, salt, and microorganisms; Johnson et al. 2003; Stockwell et al. 2012; Hamilton et al. 2012; Searle et al. 2013; Schmeller et al. 2014), detailed monitoring of the incubators ensured that the intended thermal regimes were produced and that the pathogen experienced regimes that mimicked the temperatures experienced by frogs in nature. Pre-existing knowledge (Woodhams et al. 2003, 2008; Piotrowski et al. 2004; Raffel et al. 2013; Stevenson et al. 2013) regarding the effects of constant and fluctuating thermal regimes on the growth of *Bd* provides context that would have allowed us to distinguish any anomalous results of our treatments that might have arisen from effects other than those of temperature.

Prior to all analyses, we compared the thermal data from the incubators to the natural thermal regimes at which the incubators were set. The only treatment that did not experience temperatures within one standard deviation of field temperatures (i.e., ±0.5°C from desired) was the Low-Rheocola treatment, which experienced the daily maximum temperature for ∼48 h starting on Day 4 of the experiment. Because this temperature is within the range selected by *L. rheocola* in the wild (Roznik 2013), we retained these data for analysis.

### Culture and maintenance of *Batrachochytrium dendrobatidis*

We used the *Bd* isolate Paluma-Lgenimaculata #2-2011-CO from the collection maintained at the School of Public Health, Tropical Medicine and Rehabilitation Sciences, James Cook University. The isolate was obtained from a *L. serrata* tadpole collected at Birthday Creek, near Paluma, Queensland, Australia (18°58′54″S 146°10′02″E) and had been passaged serially in vitro 18 times at the time of our experiment. The culture was maintained in TGhL broth (8 g tryptone, 1 g gelatin hydrolysate, and 2 g lactose in 1L distilled water) in 25 cm^2^ tissue culture flasks (Techno Plastic Products, Trasadingen, Switzerland) at 4°C and passaged every 2 months. When in use, the culture was maintained at 21–23°C and passaged weekly to maintain active growth. For each passage, we extracted 1 mL of active culture and added this to 9 mL of fresh TGhL in a new tissue culture flask.

### Preparation of inocula

We inoculated fifteen TGhL agar plates (TGhL as above with 10 g bacteriological agar) with 0.75–1 mL of *Bd* broth culture. Plates were sealed with Parafilm® and incubated at 21–23°C for 3 days until we observed maximum zoospore production. Zoospores were harvested by flooding the agar plates with 3 mL of TGhL broth. The zoospore suspension was vacuum filtered through a sterile 20 *μ*m nylon filter (Spectra Mesh; Spectrum Laboratories Inc, Rancho Dominquez, CA) to remove zoosporangia. The zoospore concentration was determined by counting active zoospores on a hemocytometer (Neubauer Improved Bright-line). We counted zoospores on each of five 0.005 mm^2^ squares in each of two counting chambers, and used the average count to estimate the density of zoospores per mL of culture.

The zoospores were resuspended in TGhL at a concentration of 2.0 × 10^6^ zoospores per mL prior to inoculation into twelve 96-well plates (Costar 3595, Corning, NY). Each plate was divided into five sections: Two sections (30 replicates each) comprised two different growth treatments; half contained 50 *μ*L *Bd*/50 *μ*L TGhL (1.0 × 10^6^ zoospores per mL), and the other half contained 25 *μ*L *Bd*/75 *μ*L TGhL (0.5 × 10^6^ zoospores per mL). A further two sections comprised negative controls for each concentration (six replicates each) as described for the treatment sections above, but with heat-killed *Bd* (maintained at 60°C for 45 mins). The final section (24 replicates) contained 100 *μ*L TGhL broth (used only as controls to check for media contamination). The plates were replicated using two arrangements of this design to ensure that location within the plate did not influence growth (Appendix S2). Two 96-well plates (one of each layout) were haphazardly assigned to each incubator to control for potential plate effects (Stevenson et al. 2013). We rotated the plates 180° every 24 h within the incubators to ensure that all wells were evenly exposed to incubator temperatures.

### *Batrachochytrium dendrobatidis* growth assay

Immediately after the initial plate setup and every 24 h thereafter for the duration of the experiment, we measured growth of *Bd* spectrophotometrically using a Multiskan Ascent 96/384 Plate Reader (MTX Lab Systems Incorporated, Vienna, VA) at an absorbance of 492 nm. Final daily absorbance values were obtained for each treatment on each day by subtracting the average optical density of the replicate negative controls from the average optical density of the replicate treatment wells. Our experiment was run for thirteen days (September 21-October 03 2012), at which time the cultures had outgrown the media (as demonstrated by plateauing of the optical density readings). We visually inspected the plates daily using an inverted light microscope to monitor zoospore activity and check for contamination. Contaminated wells had unusually high optical density readings accompanied by discoloration and were excluded from analysis. To provide a visual record of growth, we photographed representative samples of each concentration from each plate on the day of maximum zoospore release under an inverted light microscope at 10× magnification using a Panasonic DMC-G1K digital camera.

### Reproductive fitness of *Bd*: fecundity and growth overtime

We examined growth differences by quantifying the reproductive output of *Bd* (for cultures initiated at 1.0 × 10^6^ zoospores per mL only) when we observed maximum zoospore release. We measured zoospore production by sacrificing a single, randomly selected well from each plate daily. We removed 30 *μ*L from each sacrificed well and quantified the zoospore concentration per mL (as above in *Preparation of inocula*). Prior to sacrificing these wells, we photographed each under an inverted light microscope. We qualitatively assessed possible differences in *Bd* reproductive fitness among the thermal treatments by comparing the number of days to maximum zoospore release and the number of zoospores released at maximum production (*n* = 2 wells sacrificed daily from each treatment, precluding calculation of variation). We used data from the sacrificed wells to qualitatively compare reproductive potential.

### Statistical analysis

All statistical analyses were performed in SYSTAT version 13, and results were considered to be significant when *P* < 0.05. We tested for differences in patterns of *Bd* growth among the thermal regimes of high- and low-elevation *L. nannotis*, *L. rheocola,* and *L. serrata*, comparing each initial zoospore concentration (1.0 × 10^6^ or 0.5 × 10^6^ zoospores per mL) separately. First, we used a repeated-measures ANOVA to compare *Bd* growth patterns among treatments (species and elevation), using day as the repeated measure and optical density as the dependent variable. After confirming that the pattern of *Bd* growth overtime differed among the treatments (as evidenced by a significant interaction between treatment and day), we clarified these differences by comparing the maximum optical density values during both the logarithmic growth phase (from Day 7 of our experiment) and the stationary phase (from Day 13 of our experiment) using a two-way ANOVA with species and elevation as factors, and optical density as the dependent variable. When these ANOVAs were significant, we used Fisher's least significant difference (LSD) post hoc tests to clarify differences among treatments (Hochberg and Tamhane 1987). To determine whether *Bd* grown in fluctuating thermal regimes mimicking those of real frogs grew at different rates from *Bd* grown in constant temperatures equaling the mean of the fluctuating temperatures, we fit a regression line and a 95% prediction interval for the regression line to the results of the most complete set of *Bd* constant-temperature experiments to date (Stevenson et al. 2013). We then compared *Bd* growth rates from our experiment to this regression and prediction interval.

## Results

### Species-specific thermal regimes

The average thermal regimes of all frog species and elevations differed in the minimum and maximum temperatures reached, and in the diel pattern of temperature fluctuation (Fig. 1). Mean temperatures ranged from ∼15 to 25.5°C, which is within the optimal range for *Bd* growth and reproduction in constant-temperature environments (Stevenson et al. 2013). At low-elevation sites, average *Litoria serrata* and *L. rheocola* body temperatures were cooler than those of *L. nannotis* during the night, but reached warmer temperatures than *L. nannotis* during the day (Fig. 1); *L. serrata* reached substantially higher mean temperatures than the other two species (Fig. 1). At high-elevation sites, there was little overlap in the thermal regimes of the three species; *L. serrata* maintained the highest average body temperatures, *L. nannotis* fluctuated very little and maintained intermediate temperatures day and night, and means for *L. rheocola* were the lowest and most variable (Fig. 1). Overall, the average thermal regimes of *L. serrata* and *L. rheocola* differed strongly between high- and low-elevation sites, while those of *L. nannotis*, which spends much of its time in the water, were more similar (Fig. 1).

### Growth of *Bd* cultures under frog thermal regimes

**Figure 2:**
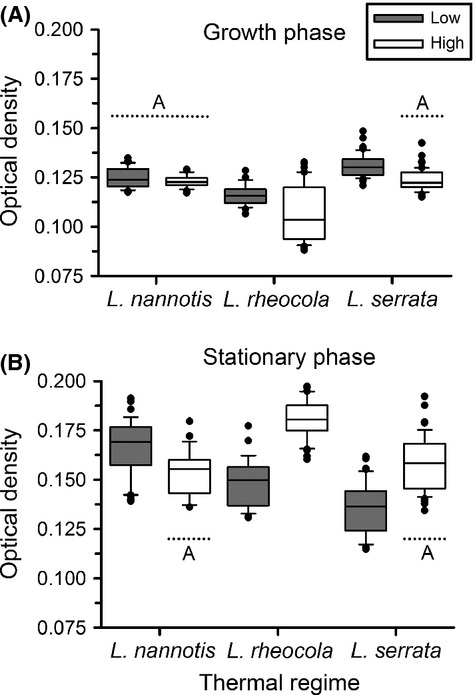
Box plots showing distributions of the optical density of *Batrachochytrium dendrobatidis* during the (A) logarithmic growth phase (Day 7 of our experiment) and (B) the stationary phase (Day 13), shown for the frogs *Litoria nannotis*, *L. rheocola*, and *L. serrata* at both low and high elevations. Horizontal lines indicate sets of temperatures that did not differ significantly; letters indicate groupings. Any temperature regime not included in a group differed significantly from all other temperature regimes for that isolate.

**Figure 3:**
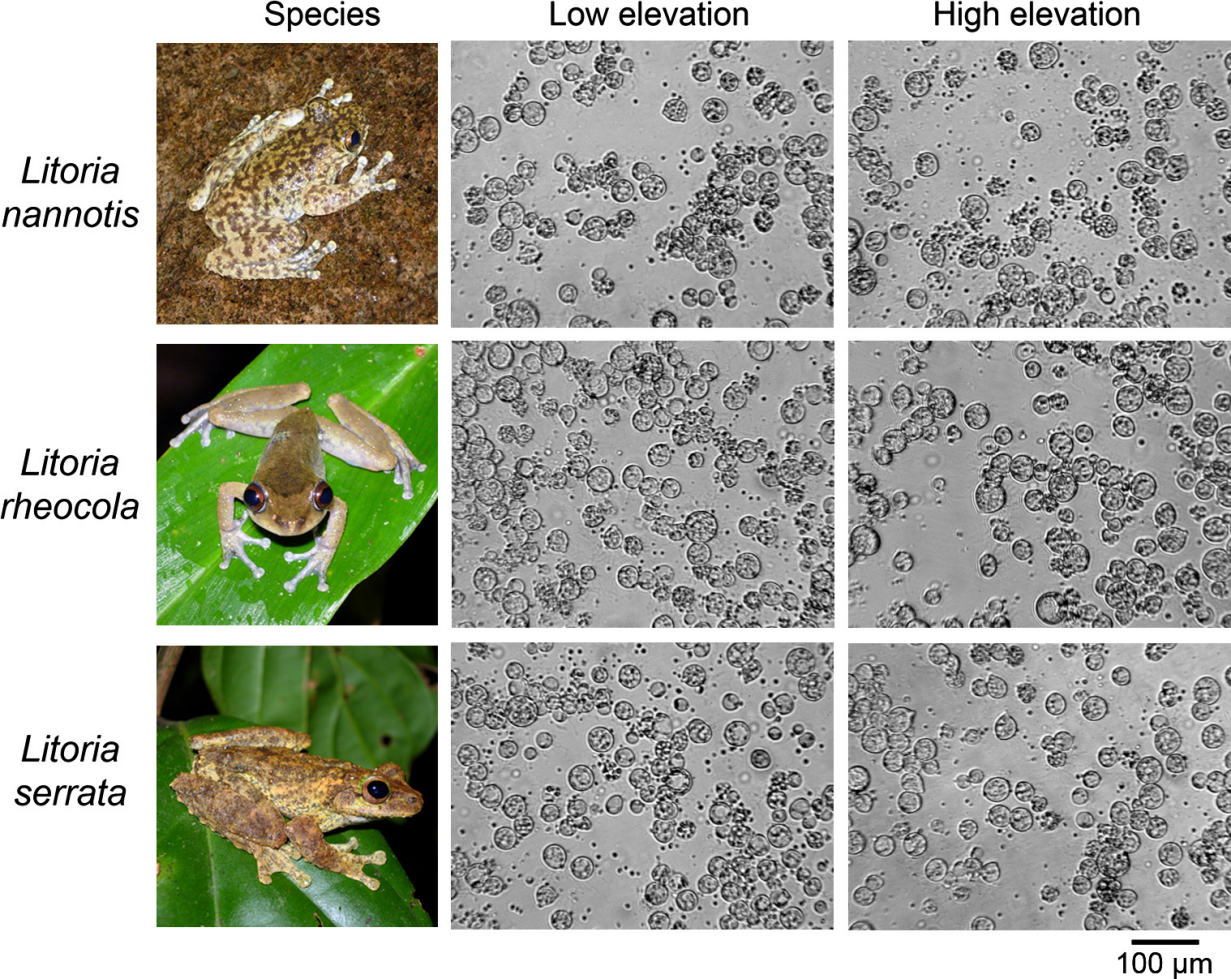
Photographs of *Batrachochytrium dendrobatidis* (*Bd*) grown under conditions representative of low and high elevations for the frogs *Litoria nannotis*, *L. rheocola*, and *L. serrata* on the day of maximum zoospore release. Initial *Bd* concentration was 1.0 × 10^6^ zoospores per mL. Frog photographs by E.A. Roznik.

Patterns of *Bd* growth over time differed significantly among replicates cultured in diel thermal regimes mimicking the average thermal regimes of *L. nannotis*, *L. rheocola,* and *L. serrata* at low and high elevations (Table 1; Figs. 2, 3). At an initial concentration of 1.0 × 10^6^ zoospores per mL, all of the thermal regimes produced very similar growth curves during the logarithmic phase; growth converged during this period and then diverged before reaching the stationary phase (Figs. 2, 3). The sole exception was that the High-Rheocola thermal regime had lower growth during the logarithmic growth phase, and continued to grow after *Bd* in all the other thermal regimes had reached the stationary phase (Figs. 2, 3). *Bd* growth differed among thermal regimes in the logarithmic growth phase on Day 7 (species: *F*_2,248_ = 83.31, *P* < 0.0001; elevation: *F*_1,248_ = 32.82, *P* < 0.0001; species × elevation: *F*_2,248_ = 4.06, *P* = 0.018; Fig. 2A) and when the cultures reached the stationary phase on Day 13 (species: *F*_2,248_ = 44.78, *P* < 0.0001; elevation: *F*_1,248_ = 71.86, *P* < 0.0001; species × elevation: *F*_2,248_ = 61.99, *P* < 0.0001; Fig. 2B). During the logarithmic growth phase, High-Nannotis thermal regimes produced optical densities that were not significantly different from Low-Nannotis (Fisher's LSD, *P* = 0.354) and were more similar to High-Serrata (*P* = 0.693), and Low-Nannotis thermal regimes were not significantly different from High-Serrata thermal regimes (*P* = 0.514; Fig. 2A). All other thermal treatments differed significantly from one another during the logarithmic growth phase (in all cases *P* < 0.002; Appendix S3).

**Table 1:** Results of repeated-measures ANOVA on effects of species, elevation, and day on growth of *Batrachochytrium dendrobatidis* in culture (initial concentration 1.0 × 10^6^ zoospores per mL).

Factor	*F*	df	*P*
Between-subjects effects			
Species	7.29	2,248	0.001
Elevation	2.08	1,248	0.150
Species × Elevation	4.55	2,248	0.011
Within-subjects effects			
Day	29,696.37	13,3224	<0.0001
Day × Species	65.05	26,3224	<0.0001
Day × Elevation	115.28	13,3224	<0.0001
Day × Species × Elevation	78.55	26,3224	<0.0001

During the stationary phase, High-Nannotis thermal regimes produced optical densities that were very similar to Low-Rheocola (*P* = 0.130) and High-Serrata (*P* = 0.139; Fig. 2B). The growth of *Bd* in the other thermal treatments differed significantly from one another during the stationary phase (in all cases *P* < 0.001; Appendix S3). As predicted, *Bd* incubated under high-elevation thermal regimes grew more under *L. rheocola* and *L. serrata* temperatures than low-elevation thermal regimes, with *L. rheocola* thermal regimes producing more growth than *L. serrata* thermal regimes. At low and high elevations, *L. nannotis* thermal regimes produced *Bd* growth that was intermediate to the responses of *L. rheocola* and *L. serrata* to low- and high-elevation thermal regimes. Counter to our prediction, *Bd* grew more at *L. nannotis* low-elevation thermal regimes than at high-elevation regimes. Results for the 0.5 × 10^6^ zoospores per mL concentration were quantitatively similar to the 1.0 × 10^6^ zoospores per mL concentration (Appendix S4).

**Figure 4:**
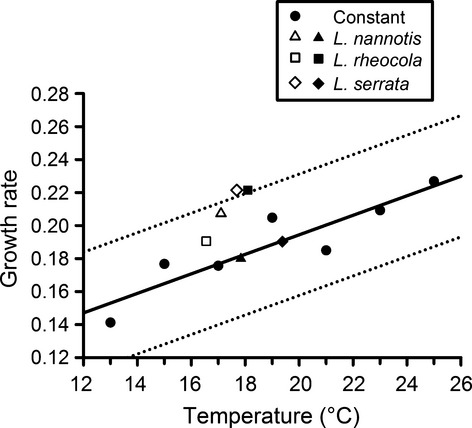
Growth rate (maximum change in optical density) of *Batrachochytrium dendrobatidis* under constant-temperature incubation (13, 15, 17, 19, 21, 23, and 25°C) (Stevenson et al. 2013) and under the fluctuating thermal regimes representing three frog species from low (closed symbols) and high (open symbols) elevations: *Litoria nannotis*, *L. rheocola*, and *L. serrata*. The maximum fluctuating regimes for each frog species and elevation were averaged throughout the day to obtain a mean overall value. The trend lines represent the predicted mean and 95% prediction intervals from a regression of the constant temperature growth data. The two fluctuating thermal regimes that are outside of the 95% prediction limits for constant temperature data are *L. serrata* from high elevations and *L. rheocola* from low elevations. Fluctuating thermal regimes are shown in Fig. 1.

Overall, growth rates of *Bd* in fluctuating frog thermal regimes were within the prediction interval derived from constant-temperature experiments, with two exceptions: The growth rates of *Bd* in the High-Serrata and Low-Rheocola thermal regimes were significantly faster than would be predicted based upon the mean of the fluctuating temperatures (i.e., above the 95% prediction limits for constant-temperature data; Fig. 4). All of the six growth rates for fluctuating thermal regimes were at or above the expected value derived from their means (Fig. 4). Our results indicate that the growth rate of *Bd* in fluctuating thermal regimes cannot be predicted by its growth rate in a constant-temperature environment that is equivalent to the mean of the fluctuating temperatures.

### Reproductive fitness: fecundity and growth overtime

**Figure 5:**
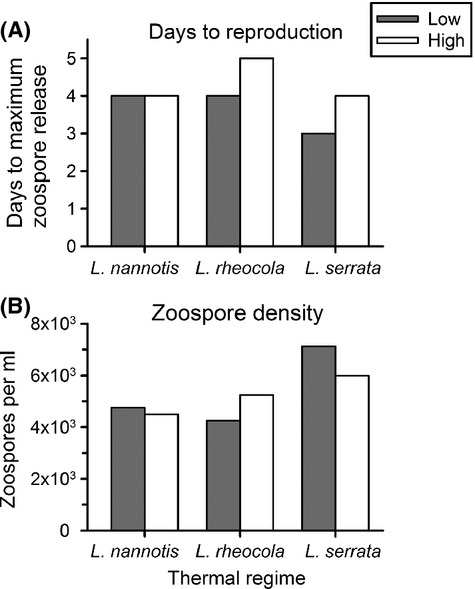
Reproductive output of *Batrachochytrium dendrobatidis*, measured as (A) days to maximum zoospore release, and (B) mean number of zoospores released (per mL) for cultures grown in the mean thermal regimes experienced by *Litoria nannotis*, *L. rheocola*, and *L. serrata* at low and high elevations. No SDs are shown because only two zoospore counts were taken per treatment. Days to maximum zoospore release were consistent within temperature treatments, and in some cases, we only had one sample per treatment for zoospore counts, precluding error bars.

The timing of zoospore production varied among frog thermal regimes, with maximum release taking place as early as Day 3 (Low-Serrata) and as late as Day 5 (High-Rheocola; Fig. 5A). *Litoria nannotis* was the only species in which maximum zoospore release occurred on the same day for both low- and high-elevation thermal regimes; for both *L. rheocola* and *L. serrata,* maximum zoospore release was a day earlier in the low-elevation thermal regimes than in the high-elevation thermal regimes (Fig. 5A). Patterns of zoospore production were somewhat different: Zoospore density showed the largest difference between low- and high-elevation thermal regimes for *L. rheocola* (19.0% difference) and *L. serrata* (15.8% difference), but was only 5.3% higher for *L. nannotis* at low-elevation thermal regimes than for *L. nannotis* at high-elevation thermal regimes (Fig. 5B).

## Discussion

In nature, host susceptibility to pathogens is affected by degree of exposure to infection, mode of transmission, and whether infected individuals recover and develop immunity (Hethcote 1976; Liu et al. 1987; deCastro and Bolker 2005); these factors can be shaped by host behavior and physiology. We found that the natural temperature regimes of three frog species had effects on *Bd* growth in culture that could largely account for observed patterns of declines in these species, which have been presumed to be caused by *Bd* infection. At low and high elevations in winter, the rainforest frogs *L. nannotis*, *L. rheocola,* and *L. serrata* maintained mean thermal regimes within the optimal range for pathogen growth (15–25°C). *Litoria serrata*, which initially declined due to *Bd* but subsequently recovered, maintained higher and more variable body temperatures than both *L. nannotis* and *L. serrata*, both of which experienced extirpation from many high-elevation sites and still have not fully recovered to their former range. Frog thermal regimes resulted in pathogen growth rates equivalent to, or significantly faster than, rates expected from constant-temperature experiments. Our results extend our knowledge of the effects of temperature on the growth of *Bd*, but also align with previous knowledge on temperature effects. Now that we understand that many of the broad ecological patterns of population declines in our focal species may be accounted for by variation in host body temperature, we can begin to understand the role of innate and adaptive immunity in mediating vulnerability to infection.

The temperatures experienced by ectothermic hosts not only influence immune function and susceptibility to infection (Rollins-Smith et al. 2002; Harris et al. 2006; Woodhams et al. 2007a; Melzer and Bishop 2010; Rowley and Alford 2013), but can also moderate the life history and fitness of pathogens. Our study species differed in their diel patterns of body temperature fluctuations, all of which, when reduced to means, were within the optimal range for *Bd* growth in uninfected frogs (Stevenson et al. 2013; Fig. 1). During cool/dry winter, when our thermal data were collected, *L. nannotis* maintained low and relatively stable body temperatures on average; this species generally selects wet microhabitats, shelters in cool damp crevices formed by boulders or is submerged underwater during the day, and when active at night prefers wet rocks and vegetation close to the stream (Hodgkison and Hero 2001; Rowley and Alford 2007a; Puschendorf et al. 2012). These microhabitats also provide stable thermal environments (Fig. 1). Mean body temperatures of *L. rheocola* were somewhat more variable; this species selects moderately dry microhabitats, typically sheltering in moist crevices between small rocks during the day and perching on vegetation during the night (Hoskin and Hero 2008; Dennis 2012; Roznik 2013). These behaviors cause individuals to maintain moderate, although variable, thermal regimes at low elevations, but result in the lowest and most variable body temperatures at high-elevation sites (Fig. 1). By contrast, mean body temperatures of *L. serrata* were the highest and most variable of the three species overall (Fig. 1). This species shelters in vegetation during the day and perches on vegetation during the night (Richards and Alford 2005; Rowley and Alford 2007a).

Based on patterns of frog species declines observed in the field, we expected that *Bd* would perform better in vitro (i.e., reach higher optical densities due to higher overall growth) in the cooler and more constant thermal regimes experienced by frogs living at high elevations than the warmer and more variable thermal regimes at low elevations. This was true for the thermal regimes of *L. rheocola* and *L. serrata*; populations of these species declined severely at high elevations, but remained stable at low elevations. *Litoria nannotis* also declined severely at high elevations, but we found the opposite pattern of pathogen performance, that is, growth was faster at thermal regimes simulating low elevations than those simulating high-elevation sites. Moisture is also important for pathogen growth and reproduction (Johnson et al. 2003), and it is possible that the wet environments selected by *L. nannotis* may influence pathogen growth differently in nature. Determining how other behavioral aspects of the microenvironment, such as moisture, interact with temperature to influence pathogen growth would be worthwhile.

Although patterns of pathogen growth matched our predictions for the thermal regimes of two of three species, we found no consistent relationship between cooler temperatures and increased pathogen development or reproductive output, counter to expectations from experiments conducted at constant temperatures (Woodhams et al. 2008; Stevenson et al. 2013) and patterns of increased prevalence and species declines in the natural environment (Richards et al. 1993; Woodhams and Alford 2005; Bielby et al. 2008). The delayed pathogen maturation, but greater zoospore production, we observed under the *L. rheocola* high-elevation regime in comparison with the *L. rheocola* low-elevation regime agreed with predictions based on life-history trade-offs (Woodhams et al. 2008), although patterns of *Bd* reproduction did not match expectations for the other two species. The thermal regimes of *L. serrata* caused *Bd* to experience the fastest maturation rate and highest zoospore production (despite predictions of reduced growth based on the persistence of *L. serrata* in the wild), whereas those of *L. nannotis* (the slowest to recover from population declines) resulted in intermediate rates of growth and reproduction. Differences in moisture preferences by these species may help to explain these patterns, for example, *L. serrata* prefers relatively dry microenvironments that limit *Bd* growth, and *L. nannotis* prefers wet microenvironments that enhance *Bd* growth, whereas *L. rheocola* prefers locations with intermediate moisture levels (Rowley and Alford 2007a, 2013; Roznik 2013).

Earlier studies on the thermal biology of *Bd* have either been conducted under constant-temperature environments (e.g., Piotrowski et al. 2004; Woodhams et al. 2008; Stevenson et al. 2013) or fluctuating thermal regimes that are not based on the thermal biology of frogs in the wild (i.e., with arbitrarily selected means and variances; Woodhams et al. 2003; Raffel et al. 2013). Our results suggest that the mean and variance of frog thermal regimes can have important effects on the growth patterns of *Bd*. In some cases, *Bd* infections can progress more slowly in fluctuating than constant thermal regimes (Woodhams et al. 2003), although they can also show accelerated growth under fluctuating conditions (Raffel et al. 2013; Fig. 5). In our study, *Bd* grew significantly faster in two thermal regimes (High-Serrata and Low-Rheocola) than predicted by constant-temperature experiments. This may in part reflect the asymmetrical nature of growth responses to temperature; if growth accelerates more when temperature increases a given amount above the mean than it decelerates when temperature decreases the same amount below the mean, then the growth rate in a fluctuating thermal environment can be higher than expected based on the mean temperature of that environment (Georges 1989). Although it fluctuated relatively moderately, High-Serrata was the warmest high-elevation regime, while the Low-Serrata thermal regime fluctuated more than any other low-elevation regime.

Our results are based on average thermal regimes of frogs that were uninfected by *Bd,* and were measured during the cool/dry winter season. The mean temperatures for all species, sites, and times of day were within the range that is regarded as optimal for *Bd* growth (Stevenson et al. 2013), although the actual thermal regimes of individual frogs are substantially more variable (Roznik 2013). This variation could have strong effects on susceptibility to infection; even short periods of time above 28°C may, for example, prevent any growth of *Bd* (Stevenson et al. 2013). Temperature is not the only aspect of amphibians that can influence disease susceptibility. Immune responses (Maniero and Carey 1997; Raffel et al. 2006; Ribas et al. 2009) and microbial assemblages and peptides on the skin (Rollins-Smith et al. 2002; Harris et al. 2006, 2009; Woodhams et al. 2007a,b; Melzer and Bishop 2010) also vary among species and change with temperature. Frogs may also alter their behavior in response to infection (Rowley and Alford 2013; Roznik 2013), further complicating the manner in which individuals can become infected and maintain their infections. Further work exploring how temperature interacts with these aspects is needed for a more comprehensive understanding of the full range of factors that predispose some species to be highly vulnerable to declines caused by chytridiomycosis.

Our study, the first conducted using the thermal regimes of frogs in nature, found that the average diel thermal regimes of three species of uninfected frogs may affect their vulnerability to becoming infected by *Bd* and may affect the final outcome of infection. Our results reinforce the fact that the thermal regimes experienced by amphibians in the wild are likely to influence the susceptibility of species and populations to *Bd* infection (Rowley and Alford 2013; Roznik 2013). Combining detailed information on frog thermal regimes with large-scale environmental predictor variables could provide a powerful method of assessing vulnerability of populations and species.

## Supporting Information

**Appendix S1.** The locations of field sites from which data on the thermal regimes of *Litoria nannotis, L. rheocola,* and *L. serrata* were collected. **Appendix S2.** The two different arrangements of 96-well plates used in our experiments: Plate A and Plate B. **Appendix S3.** Fisher's LSD post-hoc results for ANOVAs comparing standardised optical densities among frog thermal regimes (high or low elevation of *Litoria nannotis*, *L. rheocola*, and *L. serrata*) during the logarithmic growth phase (Day 7) and the stationary phase (Day 13) at an initial *Bd* concentration of 1.0 × 10^6^ zoospores per mL. **Appendix S4.** Complete statistical results for the 0.5 × 10^6^ zoospores per mL concentration used in the experiments, which are not presented in the text.

## References

[b1] Bancroft BA, Han BA, Searle CL, Biga LM, Olson DH, Kats LB (2011). Species-level correlates of susceptibility to the pathogenic amphibian fungus *Batrachochytrium dendrobatidis* in the United States. Biodivers. Conserv.

[b2] Berger L, Speare R, Daszak P, Green DE, Cunningham AA, Goggin CL (1998). Chytridiomycosis causes amphibian mortality associated with population declines in the rain forests of Australia and Central America. Proc. Natl Acad. Sci.

[b3] Bielby J, Cooper N, Cunningham AA, Garner TWJ, Purvis A (2008). Predicting susceptibility to future declines in the world's frogs. Conserv. Lett.

[b4] Boyle DG, Boyle DB, Olsen V, Morgan JAT, Hyatt AD (2004). Rapid quantitative detection of chytridiomycosis (*Batrachochytrium dendrobatidis*) in amphibian samples using real-time Taqman PCR assay. Dis. Aquat. Organ.

[b5] deCastro F, Bolker B (2005). Mechanisms of disease-induced extinction. Ecol. Lett.

[b6] Daly EW, Johnson PTJ (2011). Beyond immunity: quantifying the effects of host anti-parasite behaviour on parasite transmission. Oecologia.

[b7] Daskin JH, Alford RA, Puschendorf R (2011). Short-term exposure to warm microhabitats could explain amphibian persistence with *Batrachochytrium dendrobatidis*. PLoS One.

[b8] Daskin JH, Bell SC, Schwarzkopf L, Alford RA (2014). Cool temperatures reduce antifungal activity of symbiotic bacteria of threatened amphibians – implications for disease management and patterns of decline. PLoS One.

[b9] Daszak P, Strieby A, Cunningham AA, Longcore JE, Brown CC, Porter D (2004). Experimental evidence that the bullfrog (*Rana catesbeiana*) is a potential carrier of chytridiomycosis, an emerging fungal disease of amphibians. Herpetol. J.

[b10] Dennis AJ, Curtis LK, Dennis AJ, McDonald KR, Kyne PM, Debus SJS (2012). Common mistfrog, *Litoria rheocola*. Queensland's threatened animals.

[b11] Fisher MC, Garner TWJ, Walker SF (2009). Global emergence of *Batrachochytrium dendrobatidis* and amphibian chytridiomycosis in space, time, and host. Annu. Rev. Microbiol.

[b12] Georges A (1989). Female turtles from hot nests: is it duration of incubation or proportion of development at high temperatures that matters?. Oecologia.

[b13] Hamilton PT, Richardson JML, Anholt BR (2012). Daphnia in tadpole mesocosms: trophic links and interactions with *Batrachochytrium dendrobatidis*. Freshw. Biol.

[b14] Harris RN, James TY, Lauer A, Simon MA, Patel A (2006). Amphibian pathogen *Batrachochytrium dendrobatidis* is inhibited by the cutaneous bacteria of amphibian species. EcoHealth.

[b15] Harris RN, Brucker RM, Walke JB, Becker MH, Schwantes CR, Flaherty DC (2009). Skin microbes on frogs prevent morbidity and mortality caused by a lethal skin fungus. ISME J.

[b16] Harvell CD, Mitchell CE, Ward JR, Altizer S, Dobson AP, Ostfeld RS (2002). Climate warming and disease risks for terrestrial and marine biota. Science.

[b17] Hethcote HW (1976). Qualitative analyses of communicable disease models. Math. Biosci.

[b18] Hochberg Y, Tamhane AC (1987). Multiple comparison procedures.

[b19] Hodgkison S, Hero JM (2001). Daily behaviour and microhabitat use of the Waterfall Frog, *Litoria nannotis* in Tully Gorge, Eastern Australia. J. Herpetol.

[b20] Hoskin C, Hero JM (2008). Rainforest frogs of the wet tropics.

[b21] Johnson ML, Berger L, Philips L, Speare R (2003). Fungicidal effects of chemical disinfectants, UV light, desiccation and heat on the amphibian chytrid *Batrachochytrium dendrobatidis*. Dis. Aquat. Organ.

[b22] Kiesecker JM (2011). Global stressors and the global decline of amphibians: tipping the stress immunocompetency axis. Ecol. Res.

[b23] Langkilde T, Alford RA (2002). The tail wags the frog: harmonic radar transponders affect movement behavior in *Litoria lesueuri*. J. Herpetol.

[b24] Lips KR, Brem F, Brenes R, Reeve JD, Alford RA, Voyles J (2006). Emerging infectious disease and the loss of biodiversity in a Neotropical amphibian community. Proc. Natl Acad. Sci.

[b25] Liu W, Hethcote HW, Levin SA (1987). Dynamical behaviour of epidemiological models with nonlinear incidence rates. J. Math. Biol.

[b26] Maniero GD, Carey C (1997). Changes in selected aspects of immune function in the leopard frog, *Rana pipiens*, associated with exposure to cold. J. Comp. Physiol. B.

[b28] McDonald K, Campbell A, Alford RA (1999). A review of declining frogs in northern Queensland. Declines and disappearances of Australian frogs.

[b29] Melzer S, Bishop PJ (2010). Skin peptide defences of New Zealand frogs against chytridiomycosis. Anim. Conserv.

[b30] Piotrowski JS, Annis SL, Longcore JE (2004). Physiology of *Batrachochytrium dendrobatidis*, a chytrid pathogen of amphibians. Mycologia.

[b31] Puschendorf R, Alford RA, Hoskin CJ, Curtis LK, Dennis AJ, McDonald KR, Kyne PM, Debus SJS, Cashins S (2012). Waterfall frog, *Litoria nannotis*. Queensland's threatened animals.

[b32] Rachowicz LJ, Knapp RA, Morgan JAT, Stice MJ, Vredenburg VT, Parker JM (2006). Emerging infectious disease as a proximate cause of amphibian mass mortality. Ecology.

[b33] Raffel TR, Rohr JR, Kiesecker JM, Hudson PJ (2006). Negative effects of changing temperature on amphibian immunity under field conditions. Funct. Ecol.

[b34] Raffel TR, Romansic JM, Halstead NT, McMahon TA, Venesky MD, Rohr JR (2013). Disease and thermal acclimation in a more variable and unpredictable climate. Nat. Clim. Chang.

[b35] Reeder NMM, Pessier AP, Vrendenburg VT (2012). A reservoir species for the emerging amphibian pathogen *Batrachochytrium dendrobatidis* thrives in a landscape decimated by disease. PLoS One.

[b36] Retallick RWR, McCallum H, Speare R (2004). Endemic infection of the amphibian chytrid fungus in a frog community post-decline. PLoS Biol.

[b37] Ribas L, Li M-S, Doddington BJ, Robert J, Seidel JA, Simon J (2009). Expression profiling the temperature-dependent amphibian response to infection by *Batrachochytrium dendrobatidis*. PLoS One.

[b38] Richards SJ, Alford RA (2005). Structure and dynamics of a rainforest frog (*Litoria genimaculata*) population in northern Queensland. Aust. J. Zool.

[b39] Richards SJ, McDonald KR, Alford RA (1993). Declines in populations of Australia's endemic tropical rainforest frogs. Pac. Conserv. Biol.

[b40] Richards-Zawacki CL (2010). Thermoregulatory behaviour affects prevalence of chytrid fungal infection in a wild population of Panamanian golden frogs. Proc. R. Soc. B Biol. Sci.

[b41] Rollins-Smith LA, Doersam JK, Longcore JE, Taylore SK, Shamblin JC, Carey C (2002). Antimicrobial peptide defences against pathogens associated with global amphibian declines. Dev. Comp. Immunol.

[b42] Rowley JJL, Alford RA (2007a). Movement patterns and habitat use of rainforest stream frogs in northern Queensland, Australia: implications for extinction vulnerability. Wildlife Res.

[b43] Rowley JJL, Alford RA (2007b). Techniques for tracking amphibians: the effects of tag attachment, and harmonic direction finding versus radio telemetry. Amphib-Reptil.

[b44] Rowley JJL, Dodd CK, Alford RA (2010). Models in field studies of temperature and moisture. Amphibian ecology and conservation: a handbook of techniques.

[b45] Rowley JJL, Alford RA (2013). Hot bodies protect amphibians against chytrid infection in nature. Sci. Rep.

[b46] Roznik EA (2013).

[b47] Roznik EA, Alford RA (2014). Using pairs of physiological models to estimate temporal variation in amphibian body temperature. J. Therm. Biol.

[b48] Schloegel LM, Hero JM, Berger L, Speare R, McDonald K, Daszak P (2006). The decline of the sharp-snouted day frog (*Taudactylus acutirostris*): the first documented case of extinction by infection in a free-ranging wildlife species?. EcoHealth.

[b49] Schmeller DS, Blooi M, Martel A, Garner TWJ, Fisher MC, Azemar F (2014). Microscopic aquatic predators strongly affect infection dynamics of a globally emerged pathogen. Curr. Biol.

[b50] Searle CL, Mendelson JR, Green LE, Duffy MA (2013). Daphnia predation on the amphibian chytrid fungus and its impacts on disease risk in tadpoles. Ecol. Evol.

[b51] Stevenson LA, Alford RA, Bell SC, Roznik EA, Berger L, Pike DA (2013). Variation in thermal performance of a widespread pathogen, the amphibian chytrid fungus *Batrachochytrium dendrobatidis*. PLoS One.

[b52] Stockwell MP, Clulow J, Mahony MJ (2012). Sodium chloride inhibits the growth and infective capacity of the amphibian chytrid fungus and increases host survival rates. PLoS One.

[b54] Vrendenburg VT, Knapp RA, Tunstall TS, Briggs CJ (2010). Dynamics of an emerging disease drive large-scale amphibian population extinctions. Proc. Natl Acad. Sci.

[b56] Williams SE (2006).

[b57] Woodhams DC, Alford RA (2005). Ecology of chytridiomycosis in rainforest stream frog assemblages of tropical Queensland. Conserv. Biol.

[b58] Woodhams DC, Alford RA, Marantelli G (2003). Emerging disease of amphibians cured by elevated body temperature. Dis. Aquat. Organ.

[b59] Woodhams DC, Ardipradja K, Alford RA, Marantelli G, Reinert LK, Rollins-Smith LA (2007a). Resistance to chytridiomycosis varies among amphibian species and is correlated with skin peptide defences. Anim. Conserv.

[b60] Woodhams DC, Rollins-Smith LA, Alford RA, Simon MA, Harris RN (2007b). Innate immune defences of amphibian skin: antimicrobial peptides and more. Anim. Conserv.

[b61] Woodhams DC, Alford RA, Briggs CJ, Johnson M, Rollins-Smith LA (2008). Life-history trade-offs influence disease in changing climates: strategies of an amphibian pathogen. Ecology.

