# Supplementary material for: Host-specific thermal profiles affect fitness of a widespread pathogen
Source: Ecol Evol. 2014 Oct 3;4(21):4053–64. doi: 10.1002/ece3.1271 (PMC4242559; doi:10.1002/ece3.1271)
Supplement: Supplementary file 1 [file ece30004-4053-SD1.docx]

**APPENDICES**

**Appendix A****.** The locations of field sites from which data on the thermal regimes of *Litoria nannotis, L. rheocola,* and *L. serrata* were collected. The high elevation study site was the same for all three species (Windin Creek, Wooroonooran National Park; 17.365°S, 145.717°E, 750m). The low elevation sites differed among species; we studied *L. nannotis* at Kirrama Creek 8 in Girramay National Park (18.196°S, 145.868°E, 170m), *L. rheocola* at Frenchman Creek in Wooroonooran National Park (17.307°S, 145.922°E, 40m), and *L. serrata* at Stoney Creek in Djiru National Park (17.920°S, 146.069°E, 20m). See *Methods* for complete details on study dates and tracking techniques.


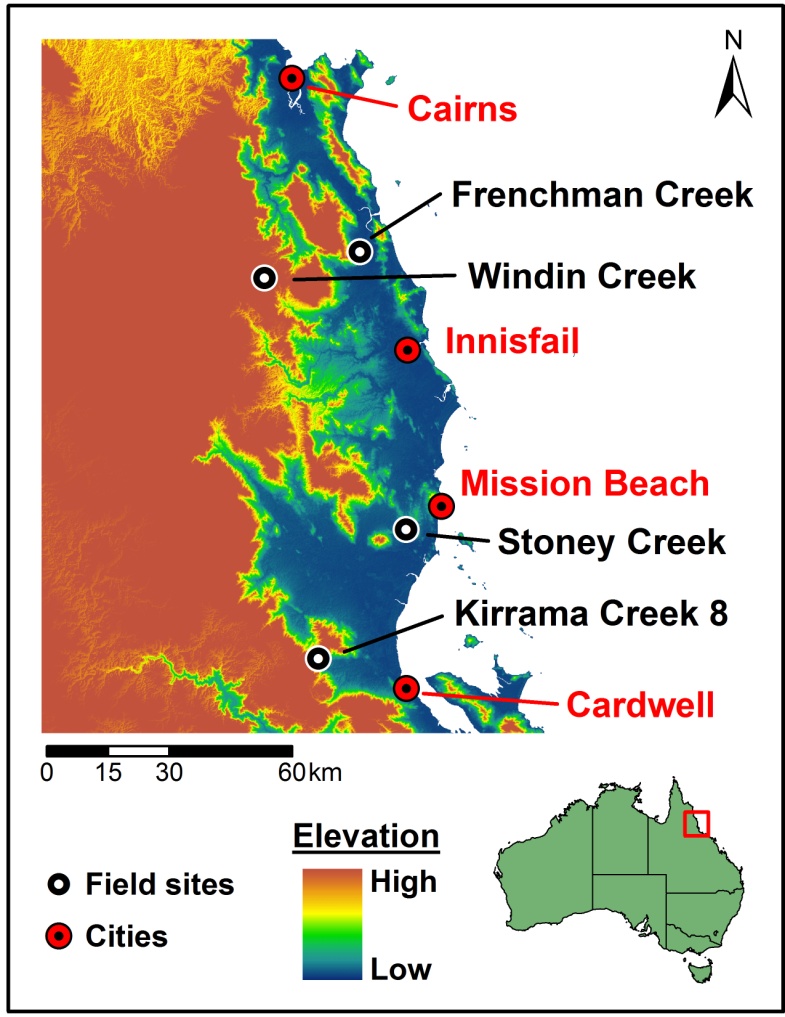
**Appendix B.** The two different arrangements of 96-well plates used in our experiments: Plate A and Plate B. One of each plate arrangement (A and B) was assigned to each thermal treatment. Each numbered column (labelled 1-12) contains eight wells arranged in rows (A-H).


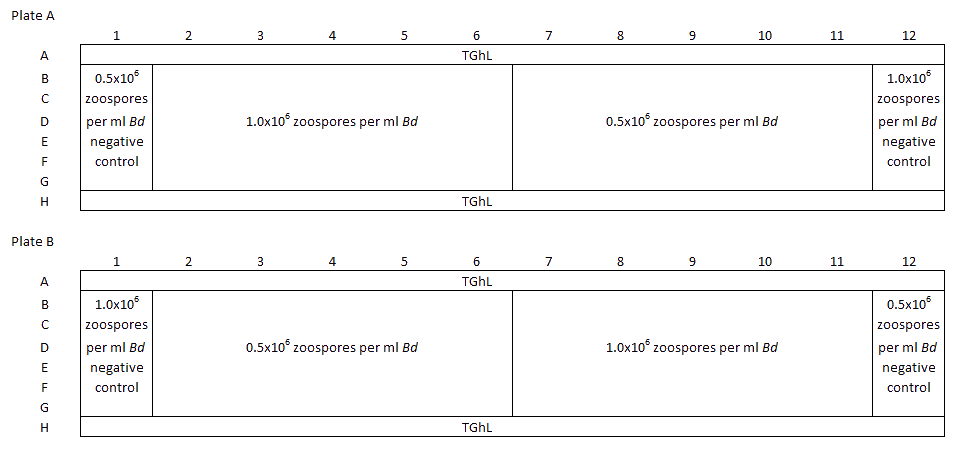


**Appendix C.** Fisher’s LSD post-hoc results for ANOVAs comparing standardised optical densities among frog thermal regimes (high or low elevation of *Litoria nannotis*, *L. rheocola*, and *L. serrata*) during the logarithmic growth phase (Day 7) and the stationary phase (Day 13) at an initial *Bd* concentration of 1.0x10^6^ zoospores per ml.

**Logarithmic growth phase (Day 7):**

|  | **Nann-Low** | **Rheo-High** | **Rheo-Low** | **Serr-High** | **Serr-Low** |
| --- | --- | --- | --- | --- | --- |
| **Nann-High** | **0.354** | <0.001 | <0.001 | **0.693** | <0.001 |
| **Nann-Low** |  | <0.001 | <0.001 | **0.514** | <0.001 |
| **Rheo-High** |  |  | <0.001 | <0.001 | <0.001 |
| **Rheo-Low** |  |  |  | <0.001 | <0.001 |
| **Serr-High** |  |  |  |  | <0.001 |

**Stationary phase (Day 13):**

|  | **Nann-Low** | **Rheo-High** | **Rheo-Low** | **Serr-High** | **Serr-Low** |
| --- | --- | --- | --- | --- | --- |
| **Nann-High** | <0.001 | <0.001 | **0.130** | **0.139** | <0.001 |
| **Nann-Low** |  | <0.001 | <0.001 | <0.001 | <0.001 |
| **Rheo-High** |  |  | <0.001 | <0.001 | <0.001 |
| **Rheo-Low** |  |  |  | <0.001 | <0.001 |
| **Serr-High** |  |  |  |  | <0.001 |

**Appendix D.** Complete statistical results for the 0.5x10^6^ zoospores per ml concentration used in the experiments, which are not presented in the text. Note that these results are similar to the 1.0x10^6^ zoospores per ml concentration, which are presented in the text.

Frog thermal regime repeated-measures ANOVA on effects of species, elevation, and day on growth of *Bd* in culture (initial concentration 0.5x10^6^ zoospores per ml).

| *Factor* | *F* | *df* | *P* |
| --- | --- | --- | --- |
| Between-subjects effects | | | |
| Species | 14.06 | 2,297 | <0.001 |
| Elevation | 61.99 | 1,197 | <0.001 |
| Species*Elevation | 46.55 | 2,297 | <0.001 |
| Within-subjects effects | | | |
| Day | 30,408.73 | 13,3861 | <0.001 |
| Day*Species | 56.45 | 26,3961 | <0.001 |
| Day*Elevation | 128.51 | 13,3861 | <0.001 |
| Day* Species*Elevation | 55.63 | 26,3961 | <0.001 |

Results for ANOVAs comparing optical densities among frog thermal regimes (high or low elevation of *Litoria nannotis*, *L. rheocola*, and *L. serrata*) during the logarithmic growth phase (Day 7) and the stationary phase (Day 13) at an initial *Bd* concentration of 25µl (0.5x10^6^ zoospores per ml).

**Logarithmic growth phase (Day 7):** species: *F*_2,297_ = 47.41, *P* < 0.0001; elevation: *F*_1,297_ = 218.01, *P* < 0.0001; species*elevation: *F*_2,297_ = 88.92, *P* < 0.0001

|  | **Nann-Low** | **Rheo-High** | **Rheo-Low** | **Serr-High** | **Serr-Low** |
| --- | --- | --- | --- | --- | --- |
| **Nann-High** | **0.739** | <0.001 | 0.001 | 0.001 | <0.001 |
| **Nann-Low** |  | <0.001 | 0.001 | 0.015 | <0.001 |
| **Rheo-High** |  |  | <0.001 | <0.001 | <0.001 |
| **Rheo-Low** |  |  |  | <0.001 | **0.942** |
| **Serr-High** |  |  |  |  | <0.001 |

**Stationary phase (Day 13):** species: *F*_2,297_ = 41.83, *P* < 0.0001; elevation: *F*_1,297_ = 19.47, *P* < 0.0001; species*elevation: *F*_2,297_ = 7.58, *P* = 0.001

|  | **Nann-Low** | **Rheo-High** | **Rheo-Low** | **Serr-High** | **Serr-Low** |
| --- | --- | --- | --- | --- | --- |
| **Nann-High** | **0.954** | 0.032 | **0.704** | 0.011 | <0.001 |
| **Nann-Low** |  | **0.059** | **0.772** | 0.034 | <0.001 |
| **Rheo-High** |  |  | <0.001 | <0.001 | 0.033 |
| **Rheo-Low** |  |  |  | **0.086** | <0.001 |
| **Serr-High** |  |  |  |  | <0.001 |
